# Orchestration of autophagosome fusion by STRIPAK complex components in muscle tissue

**DOI:** 10.1080/27694127.2023.2260670

**Published:** 2023-09-27

**Authors:** Yungui Guo, Erika R. Geisbrecht

**Affiliations:** Department of Biochemistry and Molecular Biophysics, Kansas State University, Manhattan, KS 66506, USA

**Keywords:** Autophagy, *Drosophila*, muscle, STRIPAK, Strip, NUAK

## Abstract

Autophagy is a central process responsible for the disposal of normal as well as damaged cellular proteins and organelles. Proper regulation of multiple steps – including initiation and the fusion between autophagosomes and lysosomes – is essential for the completion of cargo disposal. While the function of many proteins that mediate canonical autophagy has been characterized, the identification of new autophagy regulators may shed light on differences between tissues and/or responses to cellular stresses. In this punctum, we discuss our recent findings about how the Striatin-Interacting Phosphatase and Kinase (STRIPAK)-NUAK-Starvin (Stv) complex coordinately regulates autophagy in the muscle tissue of *Drosophila melanogaster*.

**Abbreviations:** Atg8a, autophagy-related 8a; BAG3, BCL2–associated athanogene 3; CASA, chaperone-assisted selective autophagy; Cp1, Cysteine proteinase-1; Fgop2, fibroblast growth factor receptor 1 oncogene partner 2; FLNC, Filamin C; HSC70, heat-shock cognate 70 complex; Mob4, MOB kinase activator 4; PLA, proximity ligation assay; Rab7, ras-related protein rab-7; RNAi, RNA interference; Strip, striatin-interacting protein; STRIPAK, striatin interacting phosphatase and kinase; Stv, starvin; SQSTM1, sequestosome 1; TFEB, transcription factor EB; Ub, ubiquitin.

Autophagy is a fundamental housekeeping mechanism that removes intracellular material by delivering into lysosomes. Cargo destined for lysosomal degradation may be normal cellular constituents that need to be recycled or are damaged and therefore have to be removed to prevent their accumulation. Selective autophagy broadly describes the degradation of specific cytoplasmic components that needs to be turned over. One example of a selective type of autophagy that is activated in response to mechanical tension is chaperone-assisted selective autophagy (CASA). The CASA BAG3 (BCL2–associated athanogene 3)-HSC70 (heat-shock cognate 70 complex) complex recognizes damaged client proteins, such as the actin-crosslinking protein FLNC (Filamin C). Ubiquitination of FLNC recruits the selective autophagy receptor p62/SQSTM1 (sequestosome 1) and subsequently Atg8a (autophagy-related 8a), triggering autophagosome formation and eventually leading to the lysosomal degradation of FLNC and associated components. The importance of CASA is exemplified by mutations in *BAG3* that result in childhood muscle dystrophy, cardiomyopathy, and respiratory deficiencies.

*Drosophila melanogaster* Stv (starvin), the ortholog of mammalian BAG3, biochemically and genetically interacts with the evolutionarily conserved NUAK kinase and the resulting NUAK-Stv complex plays an important role in the autophagic clearance of proteins in larval muscles. While many core components of the autophagy machinery are known, identifying additional regulators may elucidate mechanisms that underlie tissue and/or stress-specific responses that involve this pathway. Here we discuss how the NUAK-Stv complex coordinates with the striatin interacting phosphatase and kinase (STRIPAK) complex to regulate autophagy in *D. melanogaster* muscle tissues.

We first used an in silico approach to identify conserved mediators of muscle tissue maintenance [1]. Three of these candidates, Strip (striatin-interacting protein), Mob4 (MOB kinase activator 4), and Fgop2 (fibroblast growth factor receptor 1 oncogene partner 2) are part of the STRIPAK complex. Using Strip as a bait protein in *D. melanogaster* larval muscle tissue, we performed affinity purification-mass spectrometry (AP-MS) experiments and identified seven other known STRIPAK components. Interestingly, NUAK and Stv also co-purified with Strip and these physical interactions were verified in muscle tissue using the proximity ligation assay (PLA). We next employed genetic interaction assays using an RNA interference (RNAi) approach to confirm that STRIPAK complex members function with NUAK and Stv in the same biological process. These data led us to hypothesize that Strip and associated STRIPAK proteins may function in autophagy with the NUAK-Stv complex.

To assess changes in autophagy progression, we used immunostaining to detect ubiquitin (Ub), p62 and Atg8a upon RNAi knockdown of Strip in larval muscles. We observed an increased number of Ub-, p62-, or Atg8a-positive puncta in S*trip RNAi*-treated muscles compared to controls. Moreover, the percentage of puncta positive for both Ub and p62 (indicative of ubiquitinated cargo) or p62 and Atg8a (corresponding to autophagosomal precursors and autophagosomes) was also elevated in Strip knockdown muscles. This accumulation of autophagosomal intermediates suggested either increased biogenesis or a block in autophagosome turnover. We ruled out a role for Strip in the induction of autophagy as mRNA transcript levels corresponding to autophagosome (*Atg1, Atg18p*, and *p62*) or lysosome biogenesis *[TFEB transcription factor EB)* and *Cp1 (Cysteine proteinase-1)]* markers were not elevated in *Strip RNAi*-treated muscles. We next examined autophagic flux in tissue using the GFP-mCherry (mCh)-Atg8a tandem fusion protein. The GFP tag is acid-sensitive while the mCh tag is acid-insensitive. Therefore, both tags emit fluorescence resulting in yellow puncta when in autophagosomal precursors and autophagosomes. In autolysosomes, however, the GFP signal is quenched and only the red fluorescence can be detected. The Pearson correlation coefficient (PCC) was used to quantitatively assess the overlap of GFP and/or mCh as a measure of autophagosome abundance and showed increased PCC values in *Strip RNAi* muscles compared to controls. Moreover, the addition of the lysosomal inhibitor chloroquine resulted in a higher number of autophagosomes in experimental as well as control larvae expressing GFP-mCh-Atg8a, further proving that decreasing Strip function inhibits autophagy.

Two additional assays were used to confirm decreased autophagic flux after induction of *Strip RNAi* in muscle tissues. First, the full-length GFP-mCh-Atg8a fusion protein is cleaved within the lysosome during normal autophagic turnover, and the free mCh tag can be detected by Western blotting. Indeed, we observed less free mCh in *Strip RNAi* muscles. Second, since p62 gets degraded in the lysosome along with selected cargo appended with Ub, p62 levels serve as an indirect marker to assess autophagic flux. Strip depletion resulted in elevated p62 levels, further supporting the notion that autophagic flux is decreased in *Strip RNAi* muscles.

Since degradation of either the tandem fusion protein or p62 was reduced upon Strip knockdown, it is possible that lysosomal function may be compromised. However, measurements of cathepsin B proteolytic activity showed no difference between control or experimental muscles. Instead, we observed enlarged p62-positive structures directly adjacent to lysosomes, suggesting an impairment in autophagosome-lysosome fusion as the cause of the autophagic flux block observed in *Strip RNAi* muscles.

An interesting observation was the presence of enlarged Rab7 (ras-related protein rab-7)-positive structures in muscles subjected to Strip knockdown. It is known that endosomes can fuse with autophagosomes to form an intermediate structure called an amphisome, before fusion with lysosomes (Figure 1). Thus, maybe an additional role of Strip is to regulate amphisome production to ensure the complete activity of the autophagy pathway. Deciphering molecular targets of the STRIPAK complex will help solve this mystery.

**Figure 1.**
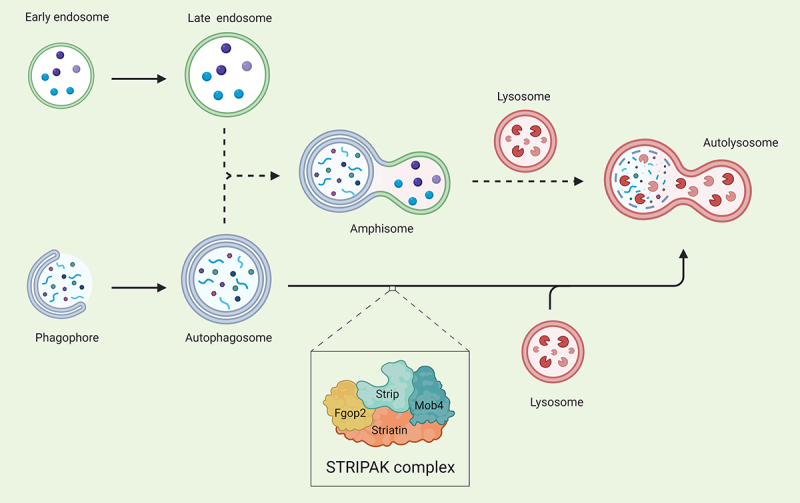
**Model depicting possible degradation routes for Strip-mediated autophagosome turnover during autophagy in *D. melanogaster* muscles**. Phagophores expand into double-membrane autophagosomes and these complete autophagosomes can directly fuse with lysosomes (solid arrows). Endosomes can also fuse with autophagosomes to produce an intermediate amphisome structure before fusion with lysosomes and subsequent cargo degradation (dotted arrows). Created with Biorender.com
